# Genome-Wide Identification and Functional Characterization of the *LbaLHCB* Gene Family Reveals Tissue-Specific Expression and Salt Stress Response in *Lycium barbarum*

**DOI:** 10.3390/ijms26199523

**Published:** 2025-09-29

**Authors:** Zhi-Hang Hu, Yue Yin, Li-Xiang Wang, Nan Zhang, Ya-Hui Wang, Jing Zhuang, Ai-Sheng Xiong

**Affiliations:** 1 State Key Laboratory of Crop Genetics & Germplasm Enhancement and Utilization, Ministry of Agriculture and Rural Affairs Key Laboratory of Biology and Germplasm Enhancement of Horticultural Crops in East China, College of Horticulture, Nanjing Agricultural University, Nanjing 210095, China; 2022204007@stu.njau.edu.cn (Z.-H.H.); wanglixiang1023@163.com (L.-X.W.); 2024204050@stu.njau.edu.cn (N.Z.); wangyahui@njau.edu.cn (Y.-H.W.); 2 National Wolfberry Engineering Research Center, Ningxia Academy of Agriculture and Forestry Sciences, Yinchuan 751002, China; yinyue2011@nwafu.edu.cn; 3 Suqian Research Institute of Nanjing Agricultural University, Facility Horticulture Research Institute of Suqian, Suqian 223800, China

**Keywords:** *Lycium barbarum*, *LHCB* gene family, genome-wide analysis, gene expression, photosynthesis regulation, salt stress response

## Abstract

The *LHCB* gene family plays a crucial role in light harvesting and photoprotection in plants by encoding key components of the photosystem II antenna complex. The *LHCB* genes are also involved in salt stress. In this study, we systematically identified and characterized 16 *LbaLHCB* genes in the economically important medicinal plant *Lycium barbarum*. Comprehensive bioinformatics analyses revealed that these genes are unevenly distributed across seven chromosomes, with notable gene clustering on chromosome 11. Phylogenetic analysis classified them into seven distinct subfamilies, with the *LbaLHCB1* subfamily showing significant expansion through gene duplication events. qRT-PCR and transcriptome analyses revealed tissue-specific expression patterns, with *LbaLHCB1.6* exhibiting preferential expression in developing fruits, suggesting its potential involvement in fruit development and quality formation. Under salt stress conditions, the *LbaLHCB* genes displayed dynamic temporal responses: *LbaLHCB1.5* was rapidly induced during early stress (1–3 h), *LbaLHCB7* reached peak expression at mid-phase (6–12 h), while *LbaLHCB1.2* showed significant downregulation during late stress response (24 h). Promoter analysis identified multiple stress-responsive *cis*-elements, providing molecular insights into their regulation under abiotic stress. These findings significantly advance our understanding of the *LbaLHCB* gene family’s structural characteristics and functional diversification in *L. barbarum*, particularly in relation to photosynthesis regulation and stress adaptation. The study provides valuable genetic resources for future molecular breeding aimed at improving stress tolerance and fruit quality in this important medicinal crop.

## 1. Introduction

Photosynthesis, the fundamental metabolic process in green plants, algae, and certain photosynthetic bacteria, converts light energy into chemical energy by synthesizing organic compounds from carbon dioxide and water while releasing oxygen [1,2]. This process occurs in chloroplasts and consists of light-dependent reactions mediated by two photosystems: photosystem I (PSI) and photosystem II (PSII) [3,4]. PSII, the initial electron donor in the photosynthetic electron transport chain, plays a pivotal role by both harvesting light energy and catalyzing water photolysis (2H_2_O → 4H^+^ + 4e^−^ + O_2_) [5,6]. This oxygen-evolving reaction represents the primary source of atmospheric oxygen, which is essential for maintaining Earth’s aerobic biosphere.

Within the intricate architecture of PSII, the light-harvesting complex II (LHCII) proteins encoded by the *Lhcb* gene family perform indispensable functions. As the peripheral antenna system of PSII, LHCII comprises multiple transmembrane proteins that efficiently bind photosynthetic pigments, including chlorophyll *a*/*b*, xanthophylls, and carotenoids [7,8]. The *Lhcb* gene family exhibits high evolutionary conservation in both sequence and structure, underscoring its fundamental role in photosynthesis. Nevertheless, *Lhcb* genes display considerable interspecies diversity, manifested through variations in gene copy number as well as species-specific expression patterns and physiological functions [9,10].

In *Arabidopsis thaliana*, the *Lhcb* gene family has been extensively characterized and classified into six members (*Lhcb1-6*), each encoding distinct subunits of the light-harvesting complex II (LHCII) with specialized roles in photosynthesis [11,12,13]. The *AtLhcb1* subfamily (including *AtLhcb1.1* to *AtLhcb1.6*) forms the core structural component of LHCII. These proteins function as the primary light-harvesting complexes, facilitating energy transfer to the PSII reaction center. Their expression levels show a strong positive correlation with photosynthetic electron transport rates, indicating a direct regulatory role in photochemical efficiency [14,15]. *AtLhcb2* and *AtLhcb3* facilitate LHCII trimerization, thereby stabilizing PSII super complexes [11]. The minor antenna proteins *AtLhcb4* (*CP29*) and *AtLhcb5* (*CP26*) not only participate in energy transfer but also play critical roles in photoprotection. Notably, *AtLhcb5* undergoes conformational changes to activate non-photochemical quenching (NPQ), mitigating photodamage under high-light stress [16,17]. Studies in crops such as tomato (*Solanum lycopersicum*) reveal that *Lhcb* genes regulate both leaf photosynthesis and fruit development, influencing yield and quality traits [18,19]. In tea plants (*Camellia sinensis*), *CsLhcb* genes exhibit diurnal oscillation patterns, with significantly higher daytime expression levels, suggesting circadian regulation of photosynthetic efficiency [20].

*Lycium barbarum* L. (wolfberry, goji berry), a perennial shrub of significant medicinal and nutritional importance, is widely cultivated in China, Southeast Asia, and parts of Europe and North America. Wolfberry is a traditional Chinese medicinal herb and is widely used as a food and dietary supplement. Its dried ripe fruit contains various bioactive compounds. Additionally, wolfberry can be eaten directly, brewed as tea, cooked in soups, or incorporated into medicinal meals, making it a classic example of a substance that serves both medicinal and dietary purposes [21]. Photosynthetic efficiency directly determines fruit quality, as optimal light exposure enhances photosynthetic rates and promotes the biosynthesis of sugars and bioactive compounds, explaining the superior quality of goji berries from high-insolation regions like Ningxia [22,23]. Fruit development in wolfberry involves complex biological processes requiring coordinated gene regulation [24,25]. Photosynthesis provides the fundamental carbon skeletons for fruit growth while simultaneously influencing the production of secondary metabolites, including carotenoids and flavonoids [26,27,28]. Despite the availability of genomic resources, the molecular mechanisms underlying photosynthesis and the functional repertoire of the *LHCB* gene family of wolfberry remain undetailed.

This study employs genomic and bioinformatic approaches to systematically identify *LHCB* family members in wolfberry, analyzing their gene structures, conserved motifs, and chromosomal distributions. Through temporal expression profiling and qRT-PCR experiments, we characterized the *LbaLHCB* gene family’s dynamic regulation during wolfberry fruit development, revealing their dual roles in maintaining photosynthetic efficiency and mediating environmental adaptation. These findings will establish a potential framework for understanding photosynthetic characteristics and inform molecular breeding strategies in wolfberry.

## 2. Results

### 2.1. Systematic Evolution Analysis of LbaLHCB Proteins in Wolfberry

**Figure 1:**
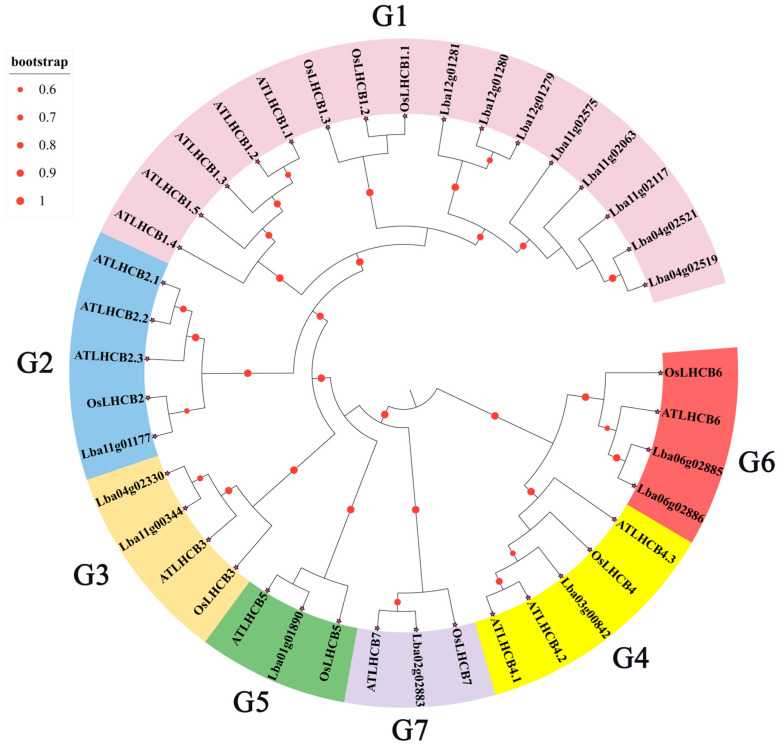
Phylogenetic trees of the *LHCB* gene in different species. Differently colored sector regions represent the seven major genomic groups (G1 to G7). The size and color intensity of the red dots indicate the corresponding bootstrap support values.

A total of 16 *LbaLHCB* family genes were identified from *Lycium barbarum* based on the reported nucleotide sequences of *LHCB* genes from *A. thaliana* and *O. sativa*. The protein sequences encoded by these genes were subjected to multiple sequence alignment using MEGA 7.0 software, and a phylogenetic tree was constructed. Phylogenetic analysis classified the LbaLHCB proteins into seven distinct subfamilies (LbaLHCB1-LbaLHCB7) based on their evolutionary relationships with homologous genes from *A. thaliana* and *O. sativa*. Each subgroup contained between 1 and 8 LbaLHCBs. Notably, the LbaLHCB1 subgroup contained multiple genes and exhibited a higher frequency of gene duplication events, suggesting its potentially significant role in adapting to diverse environmental stresses (Figure 1).

### 2.2. Analysis of Physicochemical Properties of LbaLHCB Proteins in Wolfberry

Physicochemical analysis of the LbaLHCB proteins in wolfberry (*L. barbarum*) (Table 1) revealed significant heterogeneity within this gene family. The encoded proteins exhibited a broad range of amino acid lengths (141–364 aa) and molecular weights (15.2–39.4 kDa). Theoretical isoelectric point (*pI*) data indicated that, with the exception of LbaLHCB1.1 (*pI* 9.39) and LbaLHCB1.2 (*pI* 8.59), the majority of members were acidic proteins (*pI* 4.82–7.74). Instability indices varied considerably (16.48–39.34), with LbaLHCB5 (39.34) and LbaLHCB7 (39.31) suggesting relatively high structural instability. Hydrophobicity analysis showed that the grand average of hydropathicity (GRAVY) for all proteins was near neutral (−0.057 to 0.287), while the aliphatic index was generally high (77.15–103.20), indicative of an overall hydrophobic core. LbaLHCB3.2, the largest isoform (364 aa, 39.4 kDa), exhibited distinct parameter characteristics compared to other members. Subcellular localization prediction confirmed that all 16 identified LbaLHCB family factors in wolfberry are localized to the chloroplast, consistent with their photosynthetic function.

### 2.3. Analysis of LbaLHCB Gene Structure and Conserved Motifs in Wolfberry

**Figure 2:**
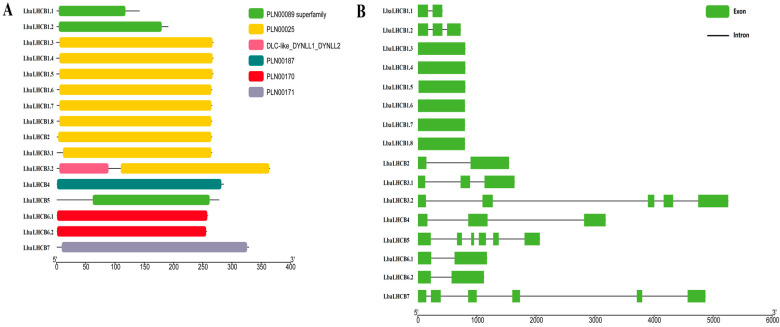
Domain and gene structure analysis of the LbaLHCB family factors in Wolfberry. (**A**) the function of the 16 kinds of Chinese wolfberry LHCB protein structure domain sequence comparison. (**B**) Genetic structure analysis of LbaLHCBs. Black lines represented intron–exon boundaries.

**Figure 3:**
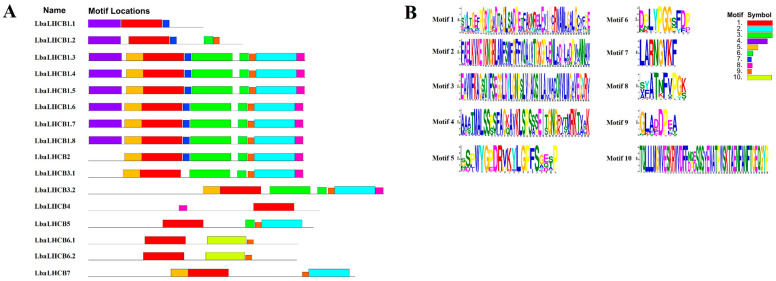
Conserved motif analysis of LbaLHCB proteins in Wolfberry. (**A**) Schematic representation of conserved protein motifs. (**B**) Legend for motif identification in Wolfberry. Motifs 1 to 10 represent different conservative motifs and are indicated by different colors. Motif 1 (red), Motif 2 (cyan), Motif 3 (green), Motif 4 (purple), Motif 5 (orange), Motif 6 (light green), Motif 7 (blue), Motif 8 (pink), Motif 9 (brown), and Motif 10 (yellow).

The protein domain composition of the *LbaLHCB* gene family members in wolfberry were also analyzed (Figure 2A). The results revealed that the 16 LbaLHCB proteins collectively contain six distinct domain types. Specifically, LbaLHCB1.1, LbaLHCB1.8, and LbaLHCB5 harbor the PLN00089 superfamily domain (indicated in green), while LbaLHCB1.3–1.8, LbaLHCB2, and LbaLHCB3.2 contain the PLN00025 family domain (indicated in yellow). The LbaLHCB3.2 possessed both the PLN00025 domain and a DLC-like_DYNLL1_DYNLL2 domain. Further analysis of gene structure (Figure 2B) demonstrated that the LbaLHCB3.2 gene is the longest (approximately 5200 bp), whereas genes encoding members LbaLHCB1.1–1.8 are all shorter than 800 bp. The LbaLHCB5 and LbaLHCB7 contain the highest number of exons (five). Conserved motifs predicted using the MEME program (Figure 3) identified ten motifs (Motif 1–10). Motif 1 is present in all members, indicating its high evolutionary conservation and likely essential functional role. Furthermore, members LbaLHCB1.3–1.8 exhibit similar conserved motif compositions, suggesting strong conservation in their protein structure and function.

### 2.4. Chromosomal Localization and Synteny Analysis of LbaLHCB Genes in Wolfberry

**Figure 4:**
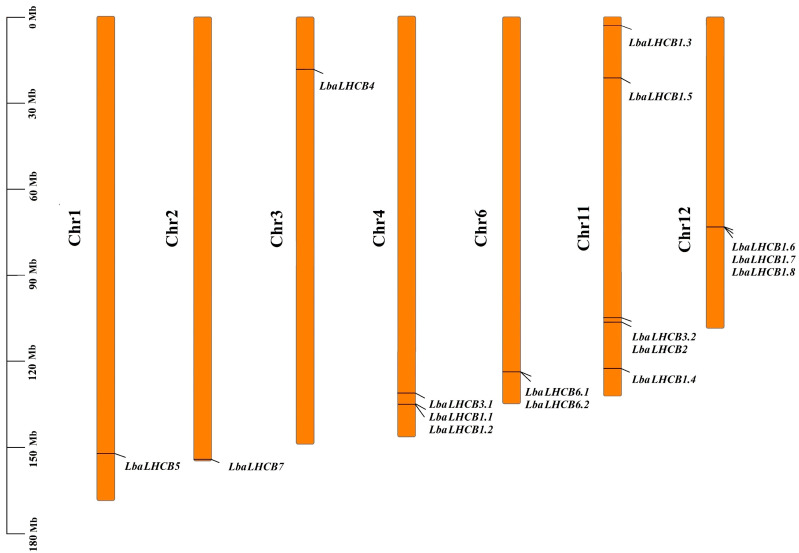
Distribution map of the *LbaLHCB* gene family on chromosomes of wolfberry.

**Figure 5:**
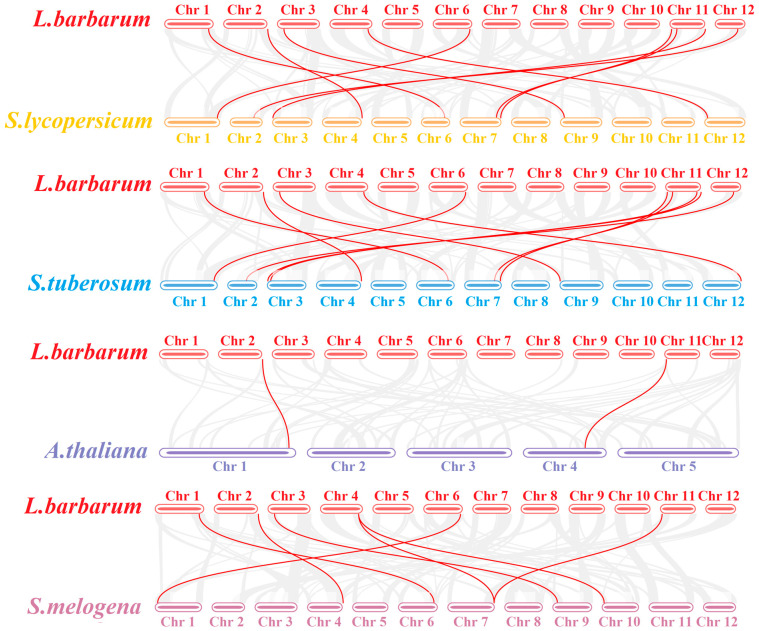
Synteny Analysis of *LHCB* Genes among Wolfberry, Tomato, Potato, *Arabidopsis*, and eggplant Genomes. Red lines represent syntenic *LbaLHCB* gene pairs.

The results presented in Figure 4 demonstrate that the *LbaLHCB* gene family exhibits an uneven distribution across seven chromosomes (Chr1, Chr2, Chr3, Chr4, Chr6, Chr11, and Chr12). The Chr11 harbors the highest gene density, containing five *LbaLHCB* genes (*LbaLHCB1.3*, *LbaLHCB1.4*, *LbaLHCB1.5*, *LbaLHCB2*, and *LbaLHCB3.2*). Three homologous gene clusters were identified: (1) *LbaLhcb1.1* and *LbaLhcb1.2* on Chr4; (2) *LbaLhcb6.1* and *LbaLhcb6.2* on Chr6; and (3) *LbaLhcb1.6*, *LbaLhcb1.7*, and *LbaLhcb1.8* on Chr12. To elucidate the evolutionary origins of the *LbaLHCB* gene family in wolfberry, we constructed syntenic maps between wolfberry and four representative species: *A. thaliana*, tomato (*S. lycopersicum*), potato (*Solanum tuberosum*), and eggplant (*Solanum melongena*) (Figure 5). The analysis revealed extensive syntenic relationships between wolfberry and the three Solanaceae species (tomato, potato, and eggplant), reflecting conserved chromosomal structures during evolution within this family. In contrast, synteny with *A. thaliana* (Brassicaceae) was limited, with only two *LHCB* homologous genes detected on the *A.* chromosomes, highlighting distinct patterns of chromosomal evolution between these plant families.

### 2.5. Analysis of Cis-Acting Elements in the Promoter Regions of LbaLHCB Genes in Wolfberry

**Figure 6:**
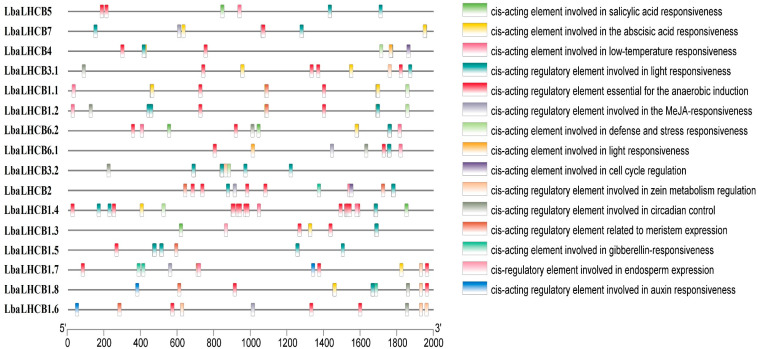
Analysis of the *cis*-acting elements of the *LbaLHCB* gene family in wolfberry.

The distribution of *cis*-acting regulatory elements was predicted within the promoter regions of *LbaLHCB* gene family members of *L. barbarum* (Figure 6). The analysis revealed that these promoter regions contain a diverse array of *cis*-acting elements implicated in responses to various environmental signals and phytohormones. Light-responsive elements were universally present across all analyzed genes, consistent with their functional role in encoding photosynthesis-related proteins. We also identified an abundance of elements associated with abscisic acid (ABA) responsiveness, methyl jasmonate (MeJA) responsiveness, salicylic acid (SA) responsiveness, and low-temperature stress responses. Additional regulatory elements were detected that participate in diverse biological processes, including: Defense and stress responses, Anaerobic induction, Auxin responsiveness, Gibberellin responsiveness, etc. This complex configuration of *cis*-acting elements suggests that *LbaLHCB* gene expression is precisely regulated by an intricate network incorporating light signaling, multiple phytohormone pathways, and abiotic stress factors, while potentially participating in specific developmental processes.

### 2.6. Heatmap Analysis of the Expression Patterns of LbaLHCBs in Different Tissues of Wolfberry

**Figure 7:**
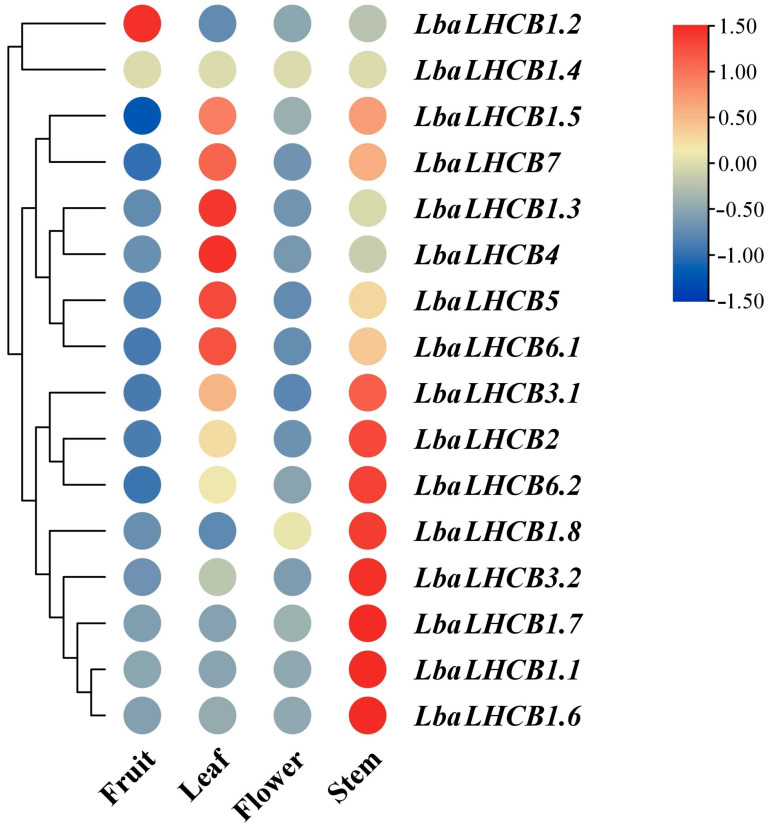
Heatmap analysis of the expression patterns of the *LbaLHCB* gene family in different tissues.

Through integrated heatmap and cluster analyses, we systematically characterized the expression patterns and functional diversification of the *LbaLHCB* gene family in wolfberry. The tissue-specific expression profiles analysis revealed distinct expression characteristics (Figure 7). *LbaLHCB1.2* demonstrated fruit-specific upregulation, implicating its involvement in fruit development processes including photosynthate accumulation and quality determination, while *LbaLHCB1.6* exhibited preferential stem expression, potentially associated with stem morphogenesis, nutrient translocation, and photosynthetic energy provision. The majority of genes (*LbaLHCB1.3* and *LbaLHCB4*) maintained moderate-to-high expression levels in leaf tissues, aligning with their fundamental roles in photosynthetic machinery. Floral organs displayed unique expression patterns, particularly for *LbaLHCB1.8*, suggesting specialized functions in floral energy metabolism and photo perceptive signaling pathways.

### 2.7. Heatmap Analysis of the Expression Patterns of LbaLHCBs Under Salt Stress of Wolfberry

**Figure 8:**
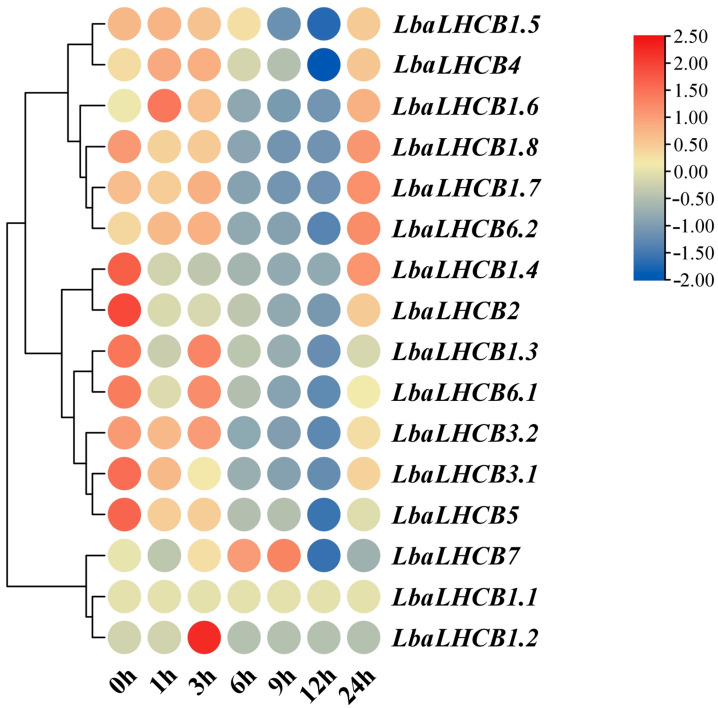
Heatmap analysis of the expression patterns of the *LbaLHCB* gene family under 300 mM NaCl stress.

Time-course analysis under 300 mM NaCl stress treatments unveiled a coordinated temporal response cascade. *LbaLHCB1.5* showed rapid induction during the early stress phase (1–3 h), indicative of its participation in initial stress perception and signaling activation. *LbaLHCB7* reached maximal expression during the intermediate phase (6–12 h), likely contributing to the maintenance of photosynthetic homeostasis under sustained stress conditions. Conversely, *LbaLHCB1.2* exhibited significant downregulation at the late stage (24 h), potentially mediating negative feedback regulation to facilitate long-term stress adaptation (Figure 8). These findings collectively demonstrate that *LbaLHCB* genes are regulated through sophisticated spatiotemporal programs, integrating developmental cues with environmental stress responses to optimize plant growth and adaptation in wolfberry.

### 2.8. Expression Characteristics of LbaLHCB Genes in Different Tissues

**Figure 9:**
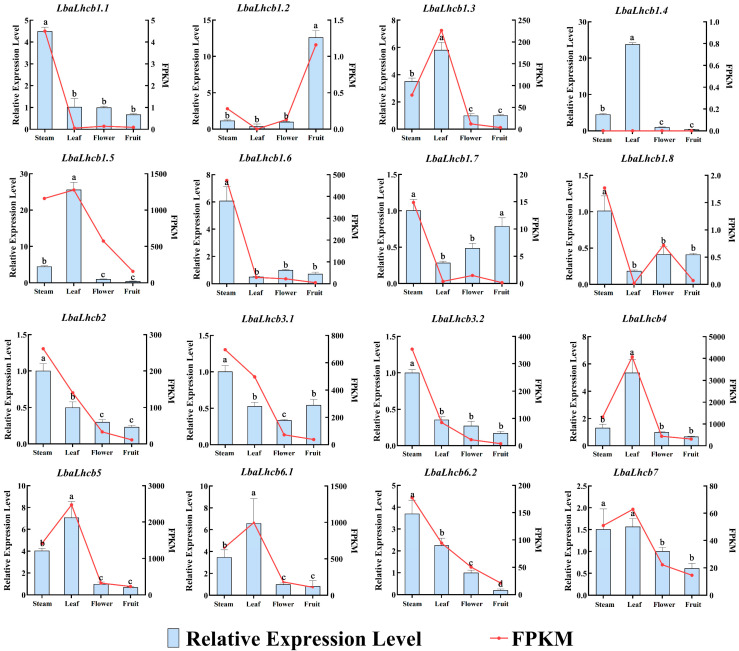
Expression patterns of *LbaLHCB* gene family in different tissues of *Lycium barbarum*. The standard deviation (SD) was represented by the error bars. Duncan multiple comparison method was used to analyze the significance of the difference between the data at the 0.05 level (*p* < 0.05). Different letters above the bars indicate statistically significant differences among tissues.

To elucidate the functional roles of the *LbaLHCB* gene family in *Lycium barbarum*, we analyzed the expression profiles of 16 *LbaLHCB* genes across stems, leaves, flowers, and fruits by integrating transcriptome sequencing data (FPKM) with qRT-PCR validation (Figure 9). The majority of genes exhibited high expression levels in leaves, such as *LbaLHCB1.3*, *LbaLHCB1.4*, *LbaLHCB4*, and *LbaLHCB5*, consistent with their essential functions in the photosystem II antenna complex. In addition, several genes showed distinct tissue-specific expression patterns. For instance, *LbaLHCB1.6* displayed markedly elevated expression in fruits, suggesting its potential involvement in fruit development and quality formation; *LbaLHCB1.2* was preferentially expressed in stems, possibly contributing to nutrient transport and energy supply; and *LbaLHCB1.8* was specifically upregulated in flowers, indicating a role in floral energy metabolism and light signal perception. Collectively, these results are consistent with the heatmap clustering analysis, confirming that most *LbaLHCB* genes are predominantly expressed in leaves, whereas certain members exhibit unique tissue-specific expression profiles.

### 2.9. qRT-PCR Validation of LbaLHCB Genes in Leaves Under Salt Stress

**Figure 10:**
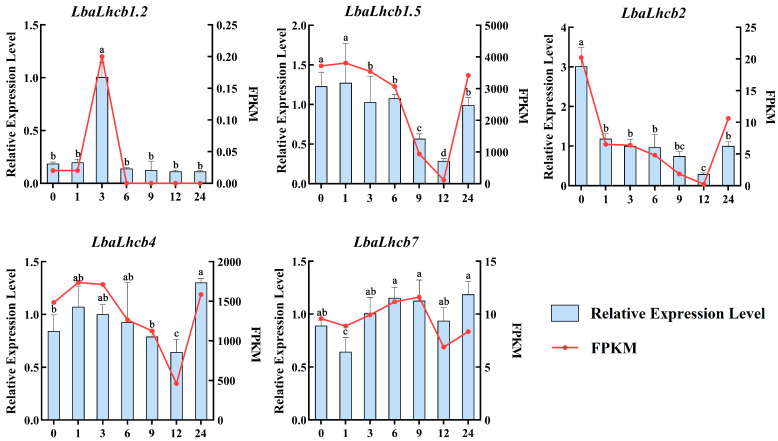
Dynamic expression profiles of *LbaLHCB* genes under salt stress treatment. The standard deviation (SD) was represented by the error bars. Duncan multiple comparison method was used to analyze the significance of the difference between the data at the 0.05 level (*p* < 0.05). Different letters above the bars indicate statistically significant differences among tissues.

To further validate the transcriptome results, five representative genes (*LbaLHCB1.2*, *LbaLHCB1.5*, *LbaLHCB2*, *LbaLHCB4*, and *LbaLHCB7*) were selected for qRT-PCR analysis based on the heatmap expression patterns. These genes displayed distinct temporal expression profiles under salt stress, which were largely consistent with the transcriptome data (FPKM), thereby supporting the reliability of the RNA-seq results (Figure 10). Specifically, *LbaLHCB1.2* was rapidly induced and peaked at 3 h before undergoing a sharp decline, suggesting its role as an early salt-responsive gene. *LbaLHCB1.5*, *LbaLHCB2*, and *LbaLHCB4* were significantly downregulated during the middle phase (6–12 h), reaching their lowest levels at 12 h, but partially recovered at 24 h, implying that they were transiently suppressed and subsequently reactivated through feedback regulation. By contrast, *LbaLHCB7* exhibited a relatively stable expression profile, characterized by a slight increase at 6–9 h, a decrease at 12 h, and a rebound at 24 h, indicating that it may contribute to the maintenance of photosystem homeostasis during prolonged salt stress.

## 3. Discussion

Wolfberry (*L. barbarum* L.), a perennial shrub with significant medicinal and nutritional value, exhibits photosynthetic efficiency that directly influences fruit quality and economic yield [29,30]. The light-harvesting complex II (LHCII) proteins, encoded by the *Lhcb* gene family, play indispensable roles in photosystem II (PSII) by facilitating light energy capture and transfer while participating in photoprotective mechanisms [31]. Here, the 16 *LbaLHCB* gene family members were systematically identified in wolfberry, suggesting potential gene expansion events during wolfberry evolution. Phylogenetic analysis classified the LbaLHCB proteins into seven distinct subfamilies (LbaLHCB1-7). The LbaLHCB1 subfamily, comprising eight members, exhibited significant gene duplication events, potentially reflecting adaptive evolution to optimize photosynthetic efficiency under diverse environmental stresses (high light intensity, drought) [9,32]. Notably, the LbaLHCB1.1 and LbaLHCB1.2 displayed basic isoelectric points (*pI* 9.39/8.59), other members were predominantly acidic (*pI* 4.82–7.74), suggesting functional diversification correlated with physicochemical properties. All members were predicted to localize to chloroplasts, consistent with their fundamental role in light harvesting [20].

Chromosomal distribution analysis revealed non-random localization of *LbaLHCB* genes, with chromosome 11 (Chr11) emerging as a genomic hotspot containing five members. Synteny analysis demonstrated extensive conservation with Solanaceae relatives (tomato, potato, and eggplant), reflecting chromosomal stability during evolution. In contrast, only two *LbaLHCB* genes showed synteny with *A. thaliana* (Brassicaceae), highlighting substantial divergence between plant families [33,34]. Tandem duplication events were observed on chromosomes 4 (LbaLhcb1.1/1.2), 6 (LbaLhcb6.1/6.2), and 12 (LbaLhcb1.6/1.7/1.8), suggesting local gene cluster expansion through subfunctionalization or neofunctionalization [35,36]. Structural analysis revealed considerable heterogeneity in domain architecture and conserved motifs. *LbaLHCB3.2*, the longest gene, contained unique domains potentially associated with specialized photosynthetic functions [37,38].

The analysis of *cis*-acting elements in the *Lhcb* gene family promoters of *Lycium barbarum* has elucidated a complex transcriptional regulatory network involving multiple signaling pathways. The promoter regions were found to contain an abundance of functionally diverse *cis*-elements, including: (i) light-responsive elements present in all *LbaLHCB* genes, which serve as binding sites for key photomorphogenic regulators (HY5, PIFs) and maintain the photoregulated expression characteristic of these photosynthetic antenna protein-encoding genes [39,40]; (ii) phytohormone-responsive elements (ABRE for ABA, MeJA- and SA-related motifs) that potentially mediate hormonal regulation of *Lhcb* expression [41]; and (iii) stress-responsive elements (MYB/MYC binding sites, DRE/CRT) that link *Lhcb* expression to abiotic stress adaptation through activation by DREB/CBF and MYB/MYC transcription factors [42,43]. The co-occurrence of these regulatory sequences indicated that there is a complex interaction among light signal transduction, hormone pathways, and stress responses in regulating the expression of the *LbaLHCB* gene. The regulation mediated by abscisic acid is particularly important for maintaining photosynthetic efficiency and implementing photoprotective mechanisms under drought and salt stress conditions [44]. These findings confirmed that the *LbaLHCB* genes possess conserved light-regulatory properties and also reveal their unique regulatory patterns in *L. barbarum*. This highlights their dual role in optimizing photosynthesis and regulating the stress adaptation of this economically important species.

With the rapid advancement of transcriptome sequencing technologies, this approach has become an indispensable tool in plant genomics research and crop genetic improvement [45,46,47]. However, current research on *L. barbarum* has primarily focused on metabolites such as carotenoids [48], with limited investigation into its responses to abiotic stresses, particularly salt stress, which remains poorly characterized [49,50]. The goji berry was regarded as a potential pioneer species for improving saline-alkali land due to their unique salt-tolerance characteristics. However, high-salt environments significantly inhibited their growth and photosynthetic efficiency. This study employed a 300 mM NaCl treatment to simulate the typical high-salt stress environment of saline-alkali land in Ningxia [51,52]. In this study, we systematically characterized the expression patterns of the *LbaLHCB* gene family in *L. barbarum* through comprehensive transcriptome analysis and qRT-PCR experiments. The results revealed temporal expression patterns that were highly consistent with the RNA-seq data. Our findings revealed that *LbaLHCB1.2* exhibited fruit-specific high expression, potentially contributing to photosynthetic product accumulation and fruit quality formation, whereas *LbaLHCB1.6* showed significant enrichment in stems, suggesting its regulatory role in stem development and energy supply. The sustained high expression levels of *LbaLHCB1.3* and *LbaLHCB4* in leaves supported their core functions within the PSII light-harvesting complex [11]. The rapid induction of *LbaLHCB1.5* within 1–3 h of salt stress coincided with the typical burst of reactive oxygen species (ROS), which served as a primary alarm signal under abiotic stress. This close temporal association suggested that *LbaLHCB1.5* functioned as an early node in the oxidative stress signaling network [53]. At the intermediate stage (6–12 h), *LbaLHCB7* expression peaked, potentially supporting photosynthetic homeostasis, a pattern similar to the co-regulation of *CsLhcb7* by circadian and stress signals in tea plants [20]. By contrast, the downregulation of *LbaLHCB1.2* at 24 h likely mediated energy reallocation, whereby plants suppressed photosynthesis-related genes to reduce investment in light harvesting and redirected resources toward defense processes such as the induction of the ion transporter SOS1 [54]. Such adjustments optimized energy use under prolonged stress and balanced metabolism with defense. Together, these patterns reflected an early–intermediate–late response model, in which light-harvesting complex (LHC) genes sequentially contributed to stress perception, photosynthetic stability, and long-term energy reallocation. Similar strategies—characterized by the downregulation of photosynthetic genes and the activation of defense genes—had been widely reported under salinity and drought stress [55].

Some evidence indicated that *Lhcb* gene expression is profoundly regulated by core circadian clock components. In tomato, CCA1 positively regulates *Lhcb1* transcription to facilitate light adaptation [19], whereas in tea plants, a skeleton photoperiod (6L6D) significantly suppresses *Lhcb* expression and delays its peak timing, consequently reducing photosynthetic efficiency [56]. The celery *Lhcb1* demonstrates broad-spectrum stress responsiveness by being induced under various adverse conditions [57], suggesting that *Lhcb* genes may serve as critical nodes integrating circadian signals and environmental stresses. These findings collectively highlight both conserved and species-specific functional specialization of the *LbaLHCB* gene family in *L. barbarum* adaptation to complex environments, underscoring their pivotal roles in plant stress responses.

In subsequent experiments, we will systematically investigate the functional roles of *LbaLHCB* genes in wolfberry’s salt tolerance mechanism through an integrated molecular-physiological approach. We will monitor photosynthetic performance by measuring key parameters using the LI-6400XT portable photosynthesis system. To comprehensively assess physiological responses, we will analyze ion homeostasis through Na+/K+ ratio determinations and evaluate water status by measuring leaf water potential and relative water content (RWC). For genes demonstrating significant stress responses, we will perform functional validation via heterologous expression in tobacco, combining detailed physiological phenotyping with molecular analyses to elucidate their specific roles in salt adaptation mechanisms.

## 4. Materials and Methods

### 4.1. Plant Materials and Treatment Methods

For salt stress response dynamic analysis, uniformly grown two-year-old *Lycium barbarum* cv. ‘Ningqi No.1’ seedlings (*n* = 60), grown at the National Wolfberry Engineering Technology Research Center in Yinchuan, Ningxia, China (37°53′ N, 106°12′ E), were subjected to treatment. Uniformly grown seedlings were subjected to salt stress by administering 300 mM NaCl solution to their root systems, increasing concentrations incrementally by 50 mM/day to prevent osmotic shock. To investigate the dynamic expression patterns of *LbaLHCB* genes in leaves under salt stress, we collected leaf samples (3rd–5th fully expanded leaves) at seven time points (0, 1, 3, 6, 9, 12, and 24 h post-treatment). Three biological replicates (individual plants) per time point were maintained under controlled conditions (25 ± 1 °C Day/18 ± 1 °C night, 60 ± 5% RH, 14 h photoperiod at 600 μmol m^−2^ s^−1^ PPFD) to ensure reproducibility. The collected samples were immediately flash-frozen in liquid nitrogen and subsequently transferred to −80 °C for storage to ensure RNA integrity for subsequent analysis.

### 4.2. Identification of LHCB Genes in the L. barbarum Genome

To identify members of the *LbaLHCB* gene family in the wolfberry (*L. barbarum*) genome, the following procedure was implemented. First, all known *A. thaliana* LHCB protein sequences were retrieved and downloaded from the TAIR database (www.arabidopsis.org/) to serve as query sequences. A BLASTP homology search was performed against the predicted protein sequence database of the *L. barbarum* genome (Accession: GCA_019175385.2) available via NCBI (www.ncbi.nlm.nih.gov/datasets/genome/?taxon=112863, accessed on 2 July 2025), using an E-value threshold of <1 × 10^−5^ to screen for potential homologous proteins. Second, the Hidden Markov Model (HMM) file for the characteristic LHCB domain (PF00504.24) was downloaded from the Pfam database (http://pfam-legacy.xfam.org/, accessed on 3 July 2025). The HMMER software (v3.3.2) package was employed to screen the predicted whole-genome protein database of *L. barbarum* (HMMER search), also using an *E*-value threshold of <1 × 10^−5^. Results from Step 1 (BLASTP) and Step 2 (HMMER) were integrated and deduplicated to generate a preliminary set of *LbaLHCB* candidate genes.

To ensure the candidate proteins encoded functional LHCB domains, conserved domain analysis was performed on all candidate protein sequences using the SMART online tool (http://smart.embl-heidelberg.de/, accessed on 3 July 2025) and the NCBI Conserved Domain Database (CDD; www.ncbi.nlm.nih.gov/cdd/, accessed on 3 July 2025). Only proteins exhibiting a clear LHCB domain in either or both databases were retained. For in-depth analysis of the physicochemical properties of the LbaLHCB proteins, molecular weight (Mw), isoelectric point (pI), and grand average of hydropathicity (GRAVY) were calculated using the ExPASy online tool (web.expasy.org/compute_pi/, accessed on 4 July 2025) and TBtools software. Subcellular localization was predicted using WoLF PSORT (wolfpsort.hgc.jp/, accessed on 4 July 2025).

### 4.3. Phylogenetic Analysis of LbaLHCB Proteins

All identified *A. thaliana* LHCB protein sequences were acquired from the TAIR database (www.arabidopsis.org/, accessed on 4 July 2025). OsLHCB family sequences were downloaded from the Joint Genome Institute database. Multiple sequence alignment was performed using MEGA 7.0 software to compare the LbaLHCB candidate protein sequences with LHCB sequences from *A. thaliana* and *O. sativa*. Based on the alignment, a phylogenetic tree was constructed using the Neighbor-Joining (NJ) method. Finally, the Interactive Tree of Life (iTOL) online platform (https://itol.embl.de/, accessed on 6 July 2025) was utilized for editing and visualization of the phylogenetic tree.

### 4.4. Gene Structure and Conserved Motif Analysis of the LbaLHCB Gene Family

The gene structure of *LbaLHCB* members was analyzed by aligning their genomic DNA sequences with corresponding cDNA sequences and visualizing the alignments using TBtools (v0.11.9). Additionally, conserved motifs in the LHCB proteins were predicted using the MEME Suite (https://meme-suite.org/meme/doc/meme.html, accessed on 6 July 2025) with the number of motifs set to 10. Identified conserved motifs were visualized using TBtools.

### 4.5. Chromosomal Localization and Synteny Analysis of LbaLHCB Gene Family Members

Chromosomal position information for the identified *LbaLHCB* gene family members was extracted from the *L. barbarum* genome annotation file (GFF3 format). A chromosomal distribution map was generated using TBtools. To investigate the evolutionary relationships within the *LbaLHCB* gene family, synteny analysis was performed between *L. barbarum* and *A. thaliana*, tomato (*S. lycopersicum*), eggplant (*Solanum melongena*), and potato (*Solanum tuberosum*) LHCB gene families using the “One Step MCScanX-Super Fast” function in TBtools. Synteny results were visualized using the “Advanced Circos” and “Multiple Synteny Plot” modules [58].

### 4.6. Cis-Acting Element Analysis of LbaLHCB Genes

Promoter sequences (2 kb upstream of the transcription start site) of *LbaLHCB* genes were extracted using TBtools. *cis*-regulatory elements within these sequences were predicted using the PlantCARE database (http://bioinformatics.psb.ugent.be/webtools/plantcare/html/, accessed on 7 July 2025). Visualization of the results was implemented using TBtools.

### 4.7. Expression Profiling of LbaLHCB Genes Based on RNA-Seq Data

To investigate the expression characteristics of the *LbaLHCB* gene family in wolfberry, RNA sequencing (RNA-seq) data for four tissues were obtained from the NCBI database (Accession: PRJNA845109) [59]. Furthermore, transcriptome sequencing was performed on leaf samples subjected to 300 mM NaCl stress treatment at specific time points (0 h, 1 h, 3 h, 6 h, 9 h, 12 h, 24 h). Based on the RNA-seq data, FPKM (Fragments Per Kilobase of transcript per Million mapped reads) expression values for the *LbaLHCB* gene family were extracted. *K*-means clustering analysis was applied to standardize expression patterns using R software (v3.2.2) [60]. Finally, clustering results were visualized using TBtools to reveal the expression trends of *LbaLHCB* genes across different time points.

### 4.8. RNA Extraction and RT-qPCR Analysis

Total RNA was extracted from tea leaves using the RNA Simple Total RNA Kit (Tiangen, Beijing, China). RNA concentration and purity were determined with a NanoDrop ND-1000 spectrophotometer (Thermo Fisher Scientific, Shanghai, China), and RNA integrity was confirmed by 1.2% agarose gel electrophoresis. qRT-PCR assays were carried out using the SYBR Premix Ex Taq Kit (TaKaRa, Dalian, China) on a Bio-Rad iQ5 real-time PCR detection system. Each 20 μL reaction contained 10 μL SYBR Green I mix, 0.4 μL of each primer (0.2 μM), 2 μL diluted cDNA, and 7.2 μL ddH_2_O. The thermal cycling program was: 95 °C for 5 min, followed by 40 cycles of 95 °C for 10 s, 54 °C for 30 s, and 65 °C for 15 s. The *LbaGAPDH* gene was used as the internal control [59], and all primers were designed using Primer Premier 5.0 software. Three biological replicates were included for each sample, and relative gene expression levels were calculated using the 2^−ΔΔCT^ method [61].

### 4.9. Data Processing and Analysis

Data were organized and sorted using Microsoft Excel 2019. Statistical significance of differences among datasets was evaluated using IBM SPSS Statistics software (version 25.0), with *p* < 0.05 considered statistically significant. Graphs were generated with GraphPad Prism 9.4 (version 9.4) to provide a visual representation of the results.

## 5. Conclusions

This study presents the first systematic characterization of the *LbaLHCB* gene family in *L. barbarum*, elucidating its genomic organization, evolutionary patterns, and functional diversification. Our findings demonstrated that the *LbaLHCB* family has undergone significant expansion through gene duplication events, particularly in the *LbaLHCB1* subfamily, which may contribute to enhanced environmental adaptability. The distinct temporal expression profiles of stress-responsive genes (*LbaLHCB1.5*, *LbaLHCB7*, and *LbaLHCB1.2*) under salt treatment revealed a sophisticated regulatory network coordinating photosynthesis and stress responses. Furthermore, the identification of fruit-preferential *LbaLHCB1.2* expression provided new insights into the molecular mechanisms underlying fruit development and quality formation. These research results have deepened our understanding of the functions of the *LHCB* genes in *L. barbarum* and have also provided valuable genetic resources for molecular breeding projects aimed at enhancing the stress resistance and improving the fruit traits of *L. barbarum*. Future studies should focus on functional validation of these candidate genes through transgenic approaches and their potential interactions with other stress-responsive pathways in *L. barbarum*.

## Figures and Tables

**Table 1:** Sequence information of *LbaLHCB* family genes in *Wolfberry*.

Gene Name	Gene Accession	Number of Amino Acid	Molecular Weight	Theoretical *pI*	Instability Index	Aliphatic Index	Grand Average of Hydropathicity	Subcellular Location
*LbaLHCB1.1*	Lba04g02519	141	15,204.67	9.39	22.49	92.84	0.15	Chloroplast
*LbaLHCB1.2*	Lba04g02521	190	20,133.58	8.59	20.06	100.68	0.287	Chloroplast
*LbaLHCB1.3*	Lba11g02117	267	28,363.39	5.47	26.45	80.04	0.023	Chloroplast
*LbaLHCB1.4*	Lba11g02063	267	28,183.19	5.46	23.64	80.45	0.042	Chloroplast
*LbaLHCB1.5*	Lba11g02575	267	28,357.31	5.47	23.13	77.15	0.015	Chloroplast
*LbaLHCB1.6*	Lba12g01281	265	28,041.94	5.14	28.11	80.34	0.045	Chloroplast
*LbaLHCB1.7*	Lba12g01279	265	28,071.96	5.15	29.15	79.25	0.027	Chloroplast
*LbaLHCB1.8*	Lba12g01278	265	28,061.92	5.15	28.5	79.25	0.03	Chloroplast
*LbaLHCB2*	Lba11g01177	265	28,742.73	5.48	27.24	78.08	−0.046	Chloroplast
*LbaLHCB3.1*	Lba04g02330	265	28,630.76	5.1	16.62	85.4	0.049	Chloroplast
*LbaLHCB3.2*	Lba11g00344	364	39,390.05	4.82	16.48	85.25	0.092	Chloroplast
*LbaLHCB4*	Lba03g00842	285	31,131.64	5.61	33.44	88.77	−0.057	Chloroplast
*LbaLHCB5*	Lba01g01890	277	29,726.22	5.71	39.34	88.52	−0.011	Chloroplast
*LbaLHCB6.1*	Lba06g02886	258	27,378.43	6.15	24.63	86.78	0.097	Chloroplast
*LbaLHCB6.2*	Lba06g02885	256	27,265.4	6.16	23.1	88.2	0.145	Chloroplast
*LbaLHCB7*	Lba02g02883	328	36,347.12	7.74	39.31	103.2	0.078	Chloroplast

## Data Availability

Data can be available upon request.
